# Joint imaging: the missing end point in pediatric von Willebrand disease trials

**DOI:** 10.1016/j.rpth.2026.107014

**Published:** 2026-10-14

**Authors:** Manuel Rodríguez López, Michael Calviño Suarez, Mauro Rubén Davoli, Ramiro Núñez Vázquez, María Teresa Álvarez Román

**Affiliations:** 1 Department of Hematology and Hemotherapy, Hospital Universitario Alvaro Cunqueiro, EOXI Vigo, Instituto de Investigación Sanitaria Galicia-Sur (IISGS), Vigo, Galiza, Spain; 2 Department of Hematology and Hemotherapy, Complejo Hospitalario Universitario A Coruña, La Coruña, Galiza, Spain; 3 Department of Hematology and Hemotherapy, Hospital Provincial del Centenario, Fundación de la Hemofilia de la provincia de Santa Fe, Argentina; 4 Department of Hematology and Hemotherapy, Hospital Universitario Virgen del Rucio, Sevilla, Spain; 5 Department of Hematology and Hemotherapy, Hospital Universitario La Paz, IdIPaz, Madrid, Spain

To the Editor,

Bergmann et al. [1] report on-demand recombinant von Willebrand factor in children with von Willebrand disease (VWD), with follow-up of up to 4 years across the phase 3 and 3b studies [1]. In a disorder this rare, pediatric follow-up of this duration is scarce and valuable.

These studies raise 1 question. The bleeds resolved, but we do not know what happened to the joints.

The reported data justify the question. Eleven of 25 patients (44%) had type 3 VWD and contributed 50 of the 104 bleeding episodes. Joints were the second most frequent bleeding site, with 17 of 104 episodes (16%), after mucosal sites. Joint bleeds also formed the largest group among episodes requiring adjunctive recombinant factor (F)VIII (9/28). One child under 6 years of age had 2 moderate bleeds into the left ankle, 1 traumatic and 1 spontaneous. One patient left the study at 10.5 months when the physician decided to start prophylaxis. Neither the reason for that decision nor whether any joint assessment was performed is reported.

The efficacy framework rested on investigator-rated hemostatic response and product consumption. It establishes whether the bleed stopped, not whether the joint returned to baseline.

The distinction matters because bleed counts alone do not describe joint disease in VWD. In the WiN cohort, arthropathy occurred in 40% of patients with verified joint bleeding compared with 10% of controls, with an impact on pain and social participation [2]. In a later analysis, patients with type 3 VWD reported fewer joint bleeds than persons with hemophilia A (HA). Despite this, their long-term joint outcome was comparable [3]. Sood et al. [4] compared 100 patients with type 3 VWD with 1814 patients with moderate HA and found no difference in loss of joint range of motion.

These 3 recent prospective VWD programs with pediatric participants recorded joint bleeds, target joints or clinical joint scores. None incorporated serial joint imaging (Table) [5], [6]. We do not intend this as criticism of the investigators. The gap extends to the frameworks that guide assessment. The European regulatory guideline does not address joint imaging [7]. The American Society of Hematology/International Society on Thrombosis and Haemostasis (ISTH)/National Hemophilia Foundation/World Federation of Hemophilia guidelines identified joint function as a critical outcome. They noted that prophylaxis studies had not reported it, nor did they make recommendations on structural or imaging assessment [8].

**Table:** Recent prospective VWD programs with pediatric participants

Study	Joint bleed information	Clinical joint assessment	Serial joint imaging
Bergmann et al. [1], phase 3/3b (rVWF)	Site, efficacy and consumption	No	No
WIL-31 (Wilate, prophylaxis) [5]	Joint ABR and target joints	HJHS longitudinally per SAP; results not published	No
WIL-33 (Wilate, prophylaxis) [6]	Joint bleeds	HJHS where age and development allowed	No

ABR, annualized bleeding rate; HJHS, Haemophilia Joint Health Score; rVWF, recombinant von Willebrand factor; SAP, statistical analysis plan.

In hemophilia, hemophilia early arthropathy detection with ultrasound (HEAD-US) has detected early structural change in asymptomatic joints, with no previous bleeds and a Haemophilia Joint Health Score of 0 [9]. We propose that future VWD trials and extension studies combine a clinical joint score with standardized ultrasound at baseline and study end, and annually in extensions, with common operator training and blinded central reading.

As an exploratory structural outcome, we propose recording the appearance or progression of cartilage or bone lesions between baseline and final scans. Changes in the total HEAD-US score and their relationship with the clinical score should also be analyzed. Before these are used as efficacy outcomes, the reproducibility of HEAD-US and the minimal detectable change in its score must be established in VWD. These definitions are not settled. Consensus is needed on which structural changes count as progression and how they should be measured in VWD. The ISTH Scientific and Standardization Committee Subcommittee on von Willebrand factor and the IPSG Imaging Expert Working Group are natural forums for this discussion.

HEAD-US was developed for hemophilic arthropathy. Its performance in VWD remains to be established [10]. It should, therefore, be assessed prospectively and in a standardized way in this population, rather than assumed valid or dismissed.

Along these lines, the Spanish Society of Thrombosis and Hemostasis (SETH) has developed a multicenter observational protocol that is awaiting ethics approval. It will compare joint health using HEAD-US in patients with type 3 VWD and patients with moderate HA (FVIII:C, 1%-5%) treated on demand. Assessments are planned at baseline and every 6 months through month 24. Findings will be correlated with the annualized joint bleeding rate, treatment modality, and quality of life. As prophylaxis is extended to younger children, joint health remains the unanswered question.
